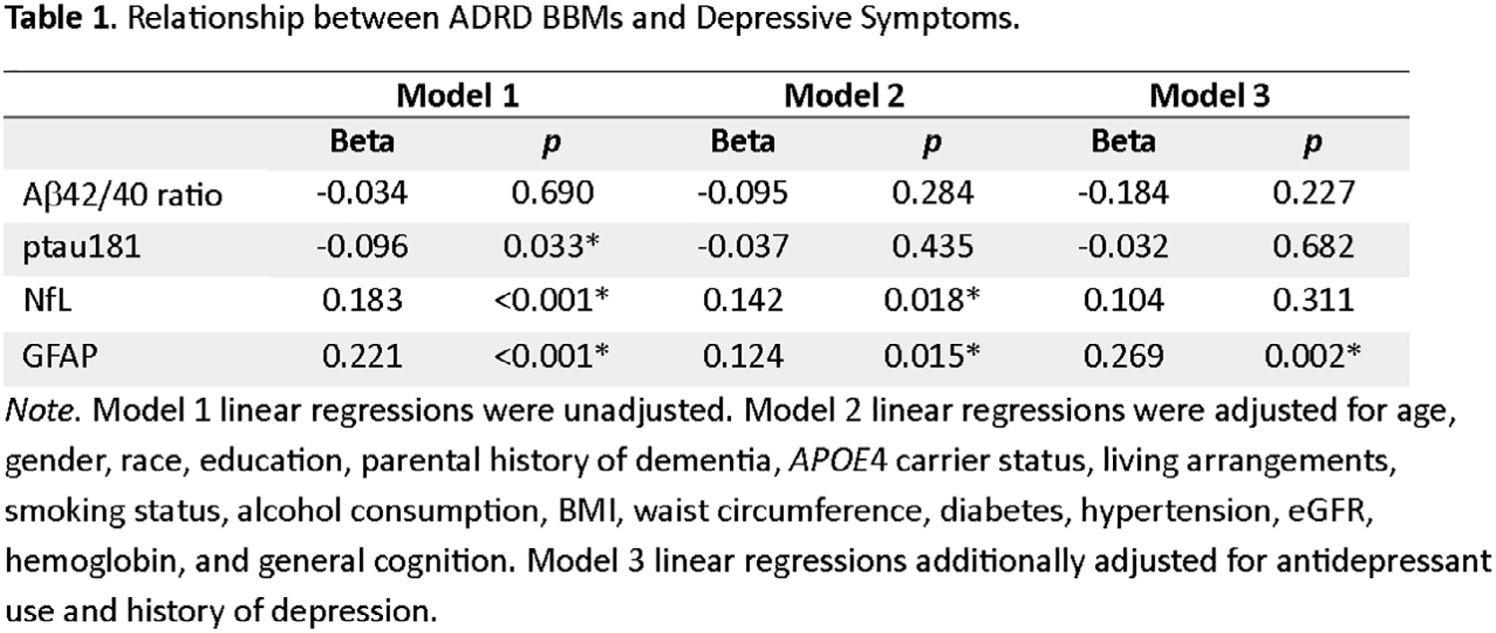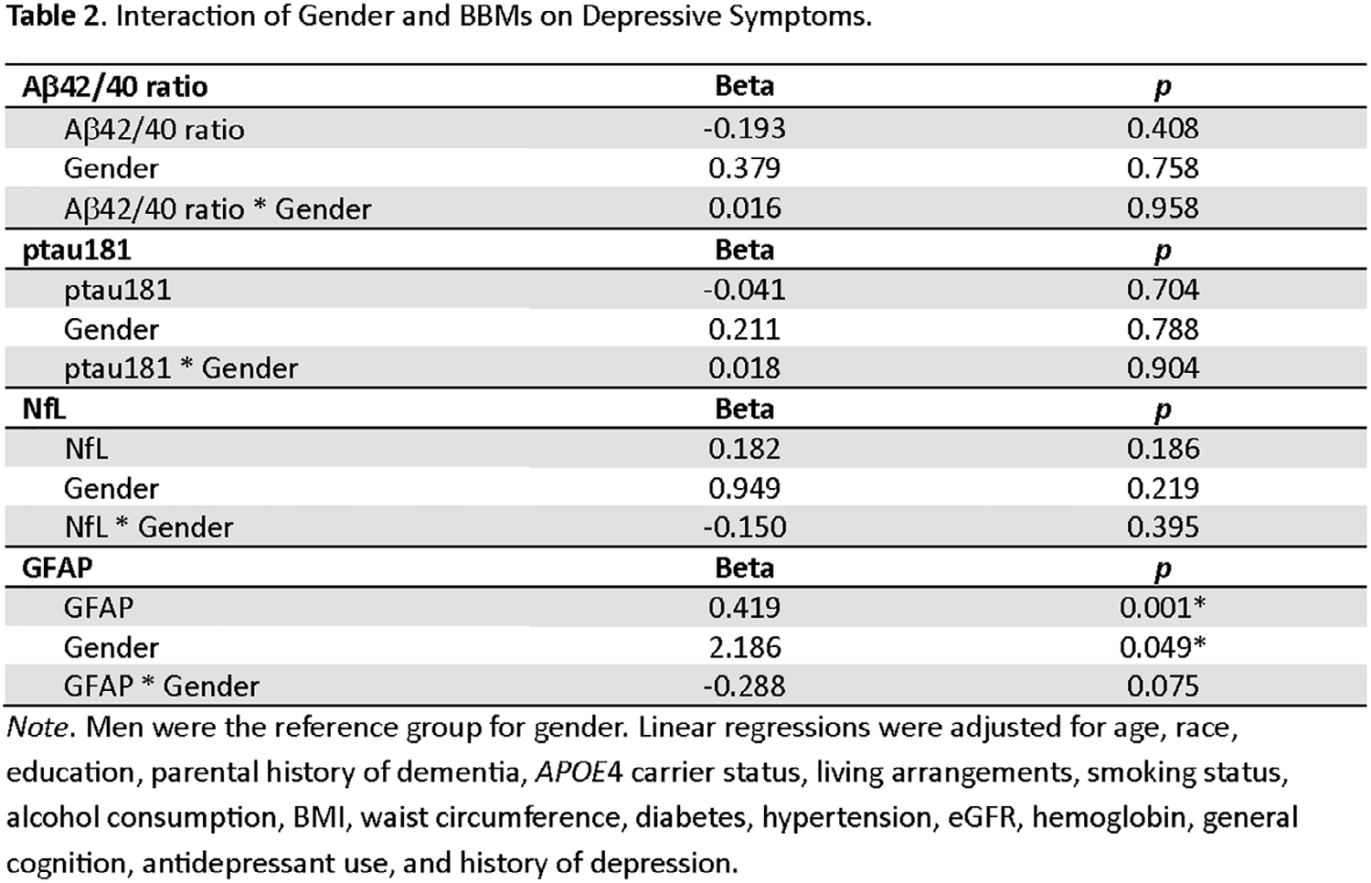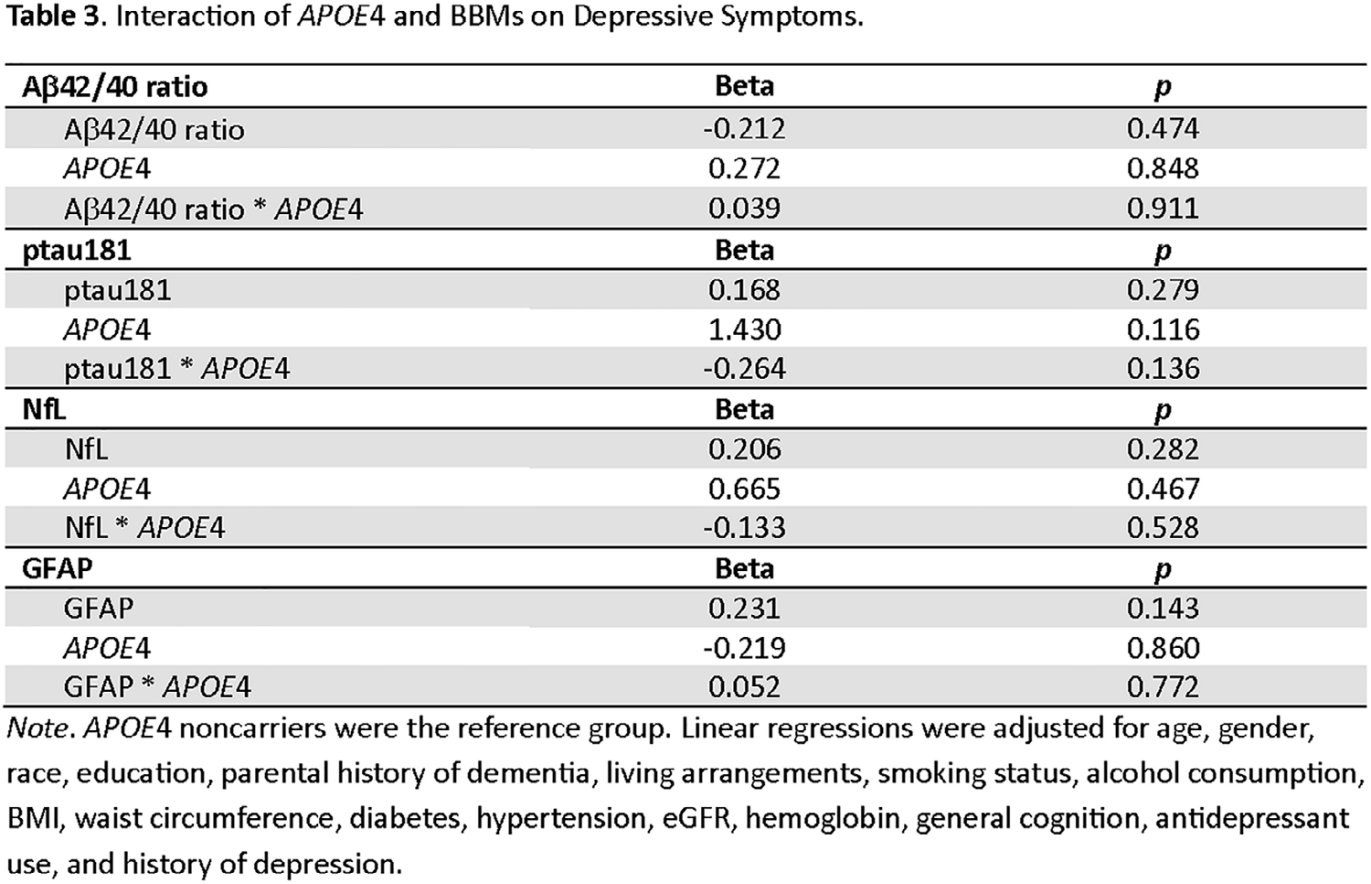# Association between Alzheimer’s Disease Blood‐Based Biomarkers and Depressive Symptoms

**DOI:** 10.1002/alz70861_108213

**Published:** 2025-12-23

**Authors:** Julia R. Bacci, Joanne Ryan, Anne Murray, Zimu Wu, Robyn L. Woods, Michael Berk, Michelle M Mielke

**Affiliations:** ^1^ Wake Forest University School of Medicine, Winston‐Salem, NC USA; ^2^ Monash University, Melbourne, VIC Australia; ^3^ Berman Center for Outcomes and Clinical Research, Hennepin Healthcare Research Institute, Minneapolis, MN USA; ^4^ Deakin University, Geelong, VIC Australia; ^5^ Division of Public Health Sciences, Wake Forest University, School of Medicine, Winston‐Salem, NC USA

## Abstract

**Background:**

Depressive symptoms are common in older adults and have been associated with both the risk for and symptoms of dementia. We examined the association between Alzheimer’s Disease and Related Dementias (ADRD) biomarkers and depressive symptoms in a large community‐based sample, taking into account cognition and a range of other participant characteristics, including BMI and kidney function which are known to influence biomarker levels. As depressive symptoms differ by gender and *APOE*4 carrier status, we also examined whether these factors modified the associations.

**Method:**

This cross‐sectional study included 11,947 non‐demented adults aged≥65 years at enrollment in ASPREE. Depressive symptoms were measured using the CESD‐10. ADRD blood‐based biomarkers (BBMs) included the Aβ42/40 ratio, ptau181, NfL, and GFAP measured on the Simoa platform. Linear regressions were used to examine associations between ADRD BBMs and depressive symptoms. Interactions between gender or *APOE*4 carrier status and BBMs were examined.

**Result:**

In unadjusted models, lower ptau181, and higher NfL or GFAP were associated with higher depressive symptoms. In multivariable models adjusting for participant characteristics, health conditions and behaviors, and cognition (see table), higher NfL and GFAP remained significantly associated with depressive symptoms. After additionally adjusting for antidepressant use and history of depression, only GFAP remained significantly associated with depressive symptoms. We did not observe significant interactions between any BBMs with gender or *APOE*4 carrier status.

**Conclusion:**

In this large, community‐based cohort of older adults, we demonstrate an association between higher plasma GFAP and greater depressive symptoms. Future research will longitudinally assess the relationship between ADRD BBMs and depressive trajectories and symptoms.